# Giant Hidrocystoma of the Orbit Presenting with Inversion and Ptosis of the Upper Eyelid

**DOI:** 10.4274/tjo.76093

**Published:** 2017-04-01

**Authors:** Melis Palamar, Banu Yaman, Taner Akalın, Ayşe Yağcı

**Affiliations:** 1 Ege University Faculty of Medicine, Department of Ophthalmology, İzmir, Turkey; 2 Ege University Faculty of Medicine, Department of Pathology, İzmir, Turkey

**Keywords:** pain, eccrine, hidrocystoma, Orbit, ptosis

## Abstract

A case of giant hidrocystoma of the orbit in a 57-year-old female causing pain, epiphora and ptosis is reported. The cystic mass was totally excised as a whole. Histopathologic examination revealed eccrine hidrocystoma of the orbit. Hidrocystoma must be considered in the differential diagnosis of patients presenting with periocular masses causing pain and ptosis.

## INTRODUCTION

Hidrocystomas are benign adnexal tumours which may be eccrine or apocrine in origin. Herein we report a case presenting with massive upper eyelid swelling and tension causing severe pain, epiphora, upper eyelid/eyelash ptosis and corneal epithelial erosion. The cyst was removed by total excision and identified as giant eccrine hidrocystoma by histopathologic examination. The aim of this case report is to serve as a reminder that this entity should be considered in the differential diagnosis of adnexal masses.

## CASE REPORT

**Figure 1:**
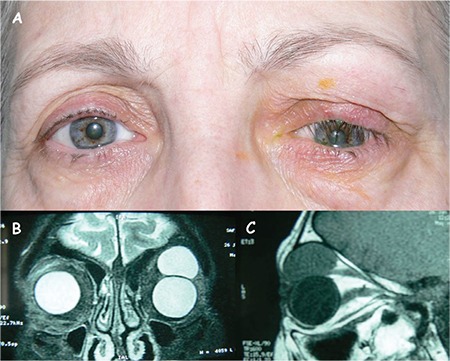
External appearance of the swollen left upper eyelid causing mechanical ptosis (A). On magnetic resonance imaging, an intraorbital, extraconal lesion is evident in both coronal T2-weighted image (B) and sagittal T1-weighted image (C)

A 57-year-old female admitted to our clinic with massive upper eyelid swelling and severe pain. On examination, eyelid and eyelash ptosis was observed due to the mechanical effect of a palpable soft, mobile mass located in the anterior orbit (Figure 1A). Inversion of eyelashes resulted, with large central corneal epithelial erosion causing irritation and epiphora. The left eye was displaced inferiorly and downgaze was partially restricted. Best corrected visual acuity was 0.5 in the affected eye. All other ophthalmologic findings were within normal limits in both eyes.

**Figure 2:**
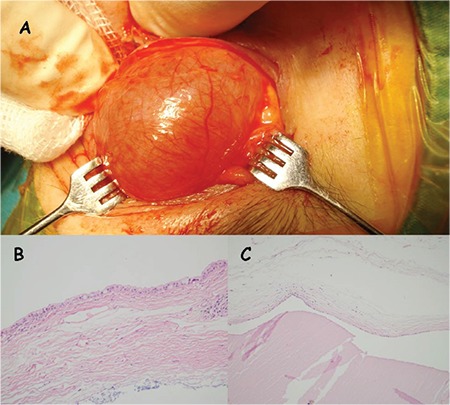
Intraoperative view demonstrating exposure and marsupialisation of the upper eyelid cyst (A). Histologic examination revealed the cyst was lined with cuboidal epithelial cells (hematoxylin and eosin x200) (B) and the cyst wall was lined with a compressed single layer of epithelial cells (hematoxylin and eosin x100) (C)

In magnetic resonance imaging, an intraorbital/extraconal large cystic lesion was observed in the superior orbit, with an anterior extension towards the eyelid (Figure 1B, 1C). A decision to excise the cystic lesion was made on the basis of clinical and radiological findings. A lid crease incision was performed and the cyst was excised as a whole using blunt dissection (Figure 2A). The wound was closed with 6/0 nylon sutures. The dimensions of the cyst were 25x20x20 mm macroscopically. Histolopathological examination revealed a cyst lined by cuboidal cells, consistent with eccrine hydrocystoma (Figure 2B). The cyst wall epithelium was compressed in most areas (Figure 2C). The postoperative outcome was good and at 12 months, magnetic resonance imaging evidenced no residual cyst.

## DISCUSSION

Hidrocystomas are benign adnexal sweat-gland lesions and are less commonly seen in the eyelids than chalasia or other benign masses.^1,2,3^ To the best of our knowledge, such cases are rarely reported in the English medical literature. The largest published series of eccrine hidrocystoma reports a mean age at diagnosis of 59 years with the majority of patients having only a single mass usually located on the upper eyelid.^1^ Periocular hidrocystomas are relatively uncommon, evidenced by the fact that they comprised only 5% of approximately 1,000 biopsied lesions in the same study. There is no gender or race predisposition.^1^ Eccrine hidrocystomas may have a similar clinical appearance to apocrine hidrocystomas. Unlike the apocrine type, which may be marginal, they tend to be distributed throughout eyelid skin without involving the eyelid margin. In addition, apocrine hidrocystomas tend to have a bluish color with yellow apical deposits. The eccrine type is more frequently associated with multiple lesions. It is rare for eccrine hidrocystoma of the eyelid to be larger than 10 mm and on average they measure 4 mm in the largest dimension.^1,2,3^ As they are considered to be ductal retention cysts, they often enlarge in perspiration-stimulating conditions such as heat and increased humidity. The eccrine hidrocystoma in our case was solitary and its dimensions were extraordinally oversized at 25x20x20 mm. Such a big cyst located in the superior part of the orbit caused posterior eyelid lamellar inversion and inferior dystopia.

Histologically, the eccrine type has a single cystic cavity, which is partially collapsed and contains no papillary projections, and lined by a one or two layers of small cuboidal epithelial cells which secrete into the glandular lumen as seen in our case (Figure 2B, 2C).^1^ The apocrine type demonstrates multiple, cystic spaces and papillary infoldings, and differs in demonstrating a fibrous outer wall of myoepithelial cells.

The differential diagnosis of the eccrine hidrocystoma includes other cystic lesions of the eyelid such as the follicular derived cysts, epidermal inclusion cyst, haemangioma, lymphangioma, apocrine hidrocystoma, and eccrine acrospiroma.^1^

Spontaneous resolution is rare, especially with large cysts, and successful management usually requires excision with complete removal of the cyst wall. Medical treatment of multiple, smaller periocular lesions has been advocated by laser thermo-ablation and, more recently, trichloroacetic acid chemical ablation.^4^ As the hidrocystoma in our case was oversized and caused additional eyelid and ocular surface problems, we chose to perform total excision.

## CONCLUSION

This rare case illustrates that ocular adnexal eccrine hidrocystoma can cause significant functional and cosmetic morbidity despite their histologically benign nature. This unusual entity should be considered in the differential diagnosis in patients presenting with periocular masses causing pain and ptosis, and may be confirmed by excision and histology.
